# Prolonged Length of Stay After Elective Same-Day Admissions for Pediatric or Congenital Cardiac Catheterizations: A Potential Measure for Procedural Harm

**DOI:** 10.1016/j.jscai.2025.103804

**Published:** 2025-08-19

**Authors:** Ralf J. Holzer, Brian Quinn, Shawn Batlivala, Lisa Bergersen, Ben Blais, Brian Boe, Lindsay F. Eilers, Howaida El-Said, Susan Foerster, Kimberlee Gauvreau, Michael Hainstock, Babar Hasan, George Nicholson, Sara M. Trucco, Michael L. O’Byrne

**Affiliations:** a UC Davis Health, Davis, California; b Boston Children's Hospital, Boston, Massachusetts; c Heart Institute, Cincinnati Children’s Hospital Medical Center, Cincinnati, Ohio; d Nationwide Children's Hospital, Columbus, Ohio; e Joe DiMaggio Children’s Hospital, Hollywood, Florida; f Texas Children’s Hospital, Houston, Texas; g Rady Children’s Hospital, San Diego, California; h Children's Wisconsin – Medical College of Wisconsin, Milwaukee Wisconsin; i UVA Health, Charlottesville, Virginia; j Sindh Institute of Urology and Transplantation (SIUT), Karachi, Pakistan; k Vanderbilt University Medical Center, Nashville, Tennessee; l UPMC Children's Hospital of Pittsburgh, University of Pittsburgh School of Medicine, Pittsburgh, Pennsylvania; m The Children's Hospital of Philadelphia, Philadelphia, Pennsylvania

**Keywords:** adverse events, cardiac catheterization, congenital heart disease, length of stay

## Abstract

**Background:**

Although adverse events (AEs) are common during congenital cardiac catheterization procedures, many have little impact beyond the catheterization laboratory. We sought to identify factors that are associated with an increased length of stay (LOS).

**Methods:**

A total of 10,882 cases from the C3PO-quality improvement registry dataset from January 2014 to December 2017 admitted electively on the same day of cardiac catheterization were analyzed and independent risk factors for a prolongation of LOS were identified.

**Results:**

Length of stay ranged from 0 to 305 days. The incidence of higher severity AE was significantly higher for cases that had a hospital stay of 2 days or more, compared to those discharged the same day or day 1 after the procedure (15% vs 2%, *P* < .001). Seven percent of patients without any AE in the cardiac catheterization laboratory had a prolonged LOS of 2 days or more. Significant independent risk factors for a prolongation of LOS included age <1 year, single ventricle diagnosis, cardiac surgery within the last 90 days, a higher hemodynamic vulnerability score, a higher PREDIC^3^T risk category, a prolonged procedure time, contrast usage >6 mL/kg, operators experience of either <5 or ≥25 years, and operator case volume >200 cases/y. The presence of any level 3bc, 4, or 5 AE had the highest associated odds of an increased LOS (OR, 5.9; 95% CI, 4.6-7.6).

**Conclusions:**

Prolonged admission after outpatient catheterization is a potential alternative measure of safety after pediatric or congenital cardiac catheterization. It is independently associated not only with patient, procedure, and operator factors that have previously been described to be associated with the risk of AE but also with other factors such as the presence of single ventricle physiology. Further studies are needed to further evaluate its utility.

## Introduction

There has been tremendous improvement in the measurement of safety in the pediatric or congenital cardiac catheterization laboratory (PCCL), to a large extent based on data and research from registries and research collaboratives such as the Congenital Cardiovascular Interventional Study Consortium and the improving pediatric and adult congenital treatment registry.^1^, ^2^, ^3^, ^4^ Despite this progress, outcome assessments are far from perfect, and continued modifications are needed. Previous research has highlighted the fact that a higher severity adverse event (AE) should not be looked at in isolation of the cardiac catheterization procedure, but needs to take into account if the cath laboratory and institution as a whole are able to successfully ameliorate the impact of such an event.^5^ “Failure to rescue” is a metric that may reflect the ability of an institution to successfully improve the ultimate outcome of a patient after a proximal AE has occurred.^5^

Our ultimate aim is to develop a metric that would differentiate between harm that extends beyond the catheterization laboratory procedure or that is recognized following a cardiac catheterization procedure without a measurable procedural adverse or inciting event, and AE that occur during a procedure, but fully resolve during the procedure itself. This would complement the existing usage of procedural AE. Reducing the incidence of high-severity AE is one way to avoid harm in PCCL patients, but it may also be possible to affect the progression from an AE to durable harm. If an AE is limited to the catheterization laboratory and has no consequence beyond the procedure, then even higher severity AE could be considered less impactful, than, for example, a low severity AE that has an associated adverse outcome beyond the procedure.

Several obstacles to creating this type of metric exist. Interpretation of the impact of AE is subjective, and to our knowledge, no standard for post-AE impact has been developed. As a step in this direction, we propose to use prolonged length of stay (LOS) after outpatient catheterization as a potential metric. Although LOS is not a direct measure of harm, it is expedient and objective. Moreover, because most patients undergoing elective cardiac catheterization are expected to be discharged within the first 24 hours of the procedure, prolonged LOS may reflect harm extending beyond the procedure and/or harm that occurred secondary to a procedure even in the absence of a measurable procedural AE.

We sought to explore the use of prolonged LOS as a metric in a multicenter retrospective cohort study using data from the C3PO registry, including the evaluation of patient- and procedure-level factors that are associated with increased likelihood of prolonged LOS. A better understanding of these characteristics is an important step to establishing prolonged LOS’s potential utility as a target for future quality improvement (QI) and research projects.

## Methods

### Data source

We analyzed data from C3PO, a collaborative of 13 centers that contributed data to this study, focused on improving outcomes of pediatric or congenital cardiac catheterization through both QI and facilitating clinical research. These data are audited yearly for accuracy and errors are corrected where identified. The protocol for the study was approved by the C3PO Research and Publications Committee. Details pertaining to registry design,^1^ AE severity level definitions,^6^ hemodynamic vulnerability,^3^ procedure type risk categories^3^^,^^7^ have previously been reported.^1^ Equally, data on the impact of a prolonged procedure length on the incidence of AE have previously been published.^8^ All physicians participating in the C3PO registry have had the opportunity to review and approve the content of this manuscript. Analysis of deidentified data does not constitute human subjects research in accordance with the Common Rule, 45 CFR 46.102(f). Because of data use agreements between member institutions, sharing of subject-level data are prohibited. The C3PO-QI dataset was chosen as opposed to the more recent C3PO-R3 dataset, as it contained data about a broader range of potential risk factors that were not collected in the most recent dataset (such as the presence of a fellow) and also contained the specific dates of admission and discharge.

### Study population

All cases captured in the C3PO (QI) registry between January 2014 and December 2017 were considered for inclusion if they met the following criteria: admission type was classified as either “elective outpatient, same day discharge,” or “elective admission or 23-hour observation,” and date of catheterization equaled the date of admission. Cases, where either date of admission, date of discharge, date of procedure, or operator were missing, were excluded, as were cases with a LOS >365 days (n = 5), due to concerns that the year of discharge may potentially have been a mistyped number. Inpatients were excluded because it was impossible to determine whether LOS was attributed to the procedure.

### Study measures

#### Outcome

Our primary outcome was increased LOS, defined as discharge being at least 2 days after the procedure. LOS was separated into 2 different categories: 0 to 1 day, and ≥2 days. The reason for this differentiation was the rationale that most patients who are admitted from home to undergo elective cardiac catheterization would be expected to be discharged the same or next day, and as such, ≥2 days was considered a possible prolongation of the expected LOS.

#### Exposures or covariates

There was not a single primary exposure. The goal of this study was to evaluate which factors were associated with prolonged LOS. Potential factors identified before analysis included nonmodifiable factors, as well as factors that are either modifiable at the institutional level or those that show important differences in how institutions provide care (Table 1).

**Table 1:** Potential predictors of an adverse outcome (increased length of stay).

Potential predictors	Discrete categories
Nonmodifiable	
Age	≤30 d, 1-11 mo, 1-17 y, and ≥18 y
Sex	Male and female
Single ventricle	Yes and no
Cardiac surgery within the last 90 days	Yes and no
Hemodynamic vulnerability	0, 1, 2, and ≥3
PREDIC^3^T risk category	1, 2, 3, 4, and 5
Highest severity adverse event	None; levels 1, 2, or 3a; and levels 3bc, 4, or 5
Modifiable at the institutional level	
Fellow present	Yes and no
Contrast usage >6 mL/kg	Yes and no
Airway management	Intubated/tracheostomy and other
Sedation	General anesthesia and other
Operator case volume	<100, 100-200, >200 cases per y
Operator experience since fellow	<5, 5-24, ≥25 y
Prolonged procedure length	Yes and no
Institutional differences	
Free-standing children’s hospital	Yes and no
Hospital cath volume	<250, 250-400, >400 cases per y

A prolonged procedure length was defined as any case where the effective procedure time exceeded 1.5× the median for the specific procedure type within the C3PO cohort (not including time for management of adverse events).

Nonmodifiable predictors included age, sex, presence of single ventricle physiology, cardiac surgery within the last 90 days, hemodynamic vulnerability, PREDIC^3^T risk category, and the presence of a high-severity AE. The definitions of hemodynamic vulnerability and PREDIC^3^T risk categories can be downloaded as a supplement to published data on the CHARM II risk-adjustment methodology.^9^

Procedural characteristics that were evaluated were the presence of a fellow, contrast usage in excess of 6 mL/kg, anesthesia method, airway management, operator case volume, operator experience since completion of the fellowship, and a prolonged procedure length, which was (uniquely) defined as a procedure length in excess of 1.5 times of the median procedure time for the specific case type (not including the time to manage an AE).

Characteristics of contributing institutions that were evaluated were the presence of a free-standing children’s hospital and the average annual catheterization volume over the study period. Annualized catheterization volume was used because it is less subject to fluctuations than the current volume and is a better reflection of exposure over time.^10^, ^11^, ^12^ Catheterization laboratory staffing mix as well as types of anesthesia providers utilized in the catheterization laboratory at institutional level were also considered as potential predictors but excluded from analysis due to insufficient variation between centers participating in the C3PO registry. Data on operator experience and the presence of a free-standing children’s hospital were obtained by contacting each participating center. Operator experience was determined at the time of each case, by calculating the time that had elapsed since completion of interventional training.

### Statistical analysis

Patient, procedural, operator, and institutional characteristics were summarized using frequencies and percentages for the entire cohort and separately for cases with 0 to 1 and ≥2 days from catheterization procedure to hospital discharge. Generalized estimating equations models evaluated the associations between each characteristic and the binary outcome ≥2 days from procedure to discharge, accounting for the clustering of patients within operators. Odds ratios are presented with 95% CIs. Multivariable analysis was performed using purposeful selection; a *P* value <.10 was required for inclusion in the final model. Analyses were performed using SAS software version 9.4 (SAS Institute).

## Results

Data from 10,882 cases performed at 13 institutions were included in this study. Basic demographic, clinical, and procedural data for the entire cohort and stratified by LOS (0-1 days, ≥2 days) is displayed in Table 2. The LOS was 1 day or less for 91% of cases, 2 days for 3% of cases, and 3 days or more for 7% of cases. About 1.4% of cases stayed 30 days or more (30-305 days). There was considerable variation between institutions, with the percentage of patients staying 2 days or more ranging from 1.9% to 12.6% (unrelated to institutional volume).

**Table 2:** Comparisons of cases with and without extended length of stay.

Characteristics	Total cohort (N = 10,882)	Days from procedure to discharge	
		0-1 d (n = 9845)	≥2 d (n = 1037)
Nonmodifiable patient and procedure characteristics			
Age			
≤30 d	63 (1%)	51 (1%)	12 (1%)
1 to 11 mo	1668 (15%)	1371 (14%)	297 (29%)
1 to 17 y	7506 (69%)	6919 (70%)	587 (57%)
≥18 y	1645 (15%)	1504 (15%)	141 (14%)
Sex			
Male	5679 (52%)	5107 (52%)	572 (55%)
Female	5086 (47%)	4629 (47%)	457 (44%)
Not recorded	117 (1%)	109 (1%)	8 (1%)
Diagnosis single ventricle	2487 (23%)	2189 (22%)	298 (29%)
Cardiac surgery within 90 d prior	318 (3%)	244 (2%)	74 (7%)
Hemodynamic vulnerability score			
0	6029 (55%)	5608 (57%)	421 (41%)
1	2345 (22%)	2164 (22%)	181 (17%)
2	1463 (13%)	1237 (13%)	226 (22%)
≥3	1045 (10%)	836 (8%)	209 (20%)
PREDIC^3^T risk category			
1	3764 (35%)	3515 (36%)	249 (24%)
2	2632 (24%)	2439 (25%)	193 (19%)
3	1944 (18%)	1720 (17%)	224 (22%)
4	1344 (12%)	1123 (11%)	221 (21%)
5	1198 (11%)	1048 (11%)	150 (14%)
Highest severity adverse event			
None	9567 (88%)	8855 (90%)	712 (69%)
Levels 1, 2, or 3a	918 (8%)	753 (8%)	165 (16%)
Levels 3bc, 4, or 5	397 (4%)	237 (2%)	160 (15%)
Potentially modifiable data			
Fellow present	908 (8%)	802 (8%)	106 (10%)
Cath procedure time above threshold			
Yes	1752 (16%)	1517 (15%)	235 (23%)
No	8237 (76%)	7504 (76%)	733 (71%)
Not recorded	893 (8%)	824 (8%)	69 (7%)
Contrast used >6 mL/kg			
Yes	1148 (11%)	910 (9%)	238 (23%)
No	8872 (82%)	8113 (82%)	759 (73%)
Not recorded	862 (8%)	822 (8%)	40 (4%)
Airway management			
Intubated or trach from the start	9723 (89%)	8780 (89%)	943 (91%)
Other	1107 (10%)	1024 (10%)	83 (8%)
Not recorded	52 (1%)	41 (<1%)	11 (1%)
Sedation			
General anesthesia	10,081 (93%)	9103 (92%)	978 (94%)
Other	738 (7%)	689 (7%)	49 (5%)
Not recorded	63 (1%)	53 (1%)	10 (1%)
Operator years since training			
<5 y	2787 (26%)	2434 (25%)	353 (34%)
5-24 y	6372 (58%)	5895 (60%)	477 (46%)
≥25 y	1723 (16%)	1516 (15%)	207 (20%)
Operator cath volume			
<100 cases/y	1945 (18%)	1784 (18%)	161 (16%)
100-200 cases/y	3984 (37%)	3710 (38%)	274 (26%)
>200 cases/y	4953 (45%)	4351 (44%)	602 (58%)
Institutional differences			
Free-standing children’s hospital	10,333 (95%)	9335 (95%)	998 (96%)
Hospital cath volume			
<250 cases/y	1564 (14%)	1434 (15%)	130 (12%)
250-400 cases/y	3849 (35%)	3532 (36%)	317 (31%)
>400 cases/y	5469 (50%)	4879 (50%)	590 (57%)

Values are n (%). Percentages provided are column percentages.

Without any procedural AE, 92.6% of patients were discharged the same or next day (7.4 of patients stayed 2 days or more). However, with any level 3bc/4/5 AE, this percentage decreased significantly to just 60%. Conversely, higher severity AE were seen in 2.4% of cases discharged the same or next day, but in 15.4% of cases discharged after 2 days or more.

The types (relationship) of AE are listed in Table 3. Most notably, out of all level 3bc/4/5 AE, 29% occurred related to valvuloplasty procedures for patients discharged 2 days or more after the procedure, but only 14% of AE were related to valvuloplasty procedures in patients discharged the same or next day after a procedure.

**Table 3:** Types of relationships of levels 3bc, 4, or 5 adverse event.

AE system	Levels 3bc, 4, or 5 AE in total cohort (N = 397)	Days from procedure to discharge	
		0-1 d (n = 237)	≥2 d (n = 160)
Valvuloplasty	81 (20%)	34 (14%)	47 (29%)
Stent	66 (17%)	46 (19%)	20 (13%)
Cath	59 (15%)	32 (14%)	27 (17%)
Device	42 (11%)	26 (11%)	16 (10%)
Sedation or airway	39 (10%)	25 (11%)	14 (9%)
Access	31 (8%)	20 (8%)	11 (7%)
Angioplasty	30 (8%)	22 (9%)	8 (5%)
Biopsy	22 (6%)	17 (7%)	5 (3%)
Coil	10 (3%)	8 (3%)	2 (1%)
Not reported	17 (4%)	7 (3%)	10 (6%)

Values are n (%).

AE, adverse event.

### Predictors of an increased LOS

Table 4 displays univariate models for potential predictors of an increased LOS of 2 days or more. In multivariable analysis (Table 5), independent predictors for an increased LOS of 2 days or more included age <1 year (OR, 2.3; 95% CI, 1.9-2.8), single ventricle diagnosis (OR, 1.5; 95% CI, 1.2-1.8), cardiac surgery within the last 90 days (OR, 1.3; 95% CI, 0.99-1.7), higher hemodynamic vulnerability score (score 2: OR, 1.8; 95% CI; 1.4-2.3; score ≥3: OR, 2.4; 95% CI, 1.7-3.4), a higher PREDIC^3^T risk category (category 3: OR, 1.3; 95% CI; 1.0-1.6; category 4: OR, 1.6; 95% CI, 1.2-2.1; category 5: OR, 1.5; 95% CI, 1.1-2.1), a procedure time above the threshold (OR, 1.5; 95% CI, 1.2-1.9), contrast usage >6 mL/kg (OR, 1.3; 95% CI, 1.0-1.6), operators experience of either <5 (OR 1.5; 95% CI, 1.2-2.0) or ≥25 years (OR, 1.7; 95% CI, 1.3-2.1) since training, operator case volume >200 cases/y (OR, 1.7; 95% CI, 1.2-2.4), and any level 3bc, 4, or 5 AE (OR, 5.9; 95% CI, 4.6-7.6).

**Table 4:** Univariate analysis for outcome: days from procedure to discharge ≥2 days.

Variables	Odds ratio	95% CI	*P* value
Age at catheterization <1 y	2.5	2.1-3.0	<.001
Sex			
Male	1.1	0.99-1.3	.067
Not recorded	0.7	0.35-1.6	.44
Diagnosis single ventricle	1.4	1.2-1.6	<.001
Cardiac surgery within 90 d prior	3.0	2.2-4.1	<.001
Hemodynamic vulnerability score (vs 0)			
1	1.1	0.93-1.3	.24
2	2.4	2.0-3.0	<.001
≥3	3.3	2.6-4.4	<.001
PREDIC^3^T risk category (vs 1)			
2	1.1	0.90-1.4	.31
3	1.8	1.5-2.3	<.001
4	2.8	2.2-3.6	<.001
5	2.0	1.5-2.8	<.001
Any levels 3bc, 4, or 5 AE	7.4	5.9-9.3	<.001
Fellow present	1.3	0.91-1.8	.15
Cath procedure time above threshold (vs no)			
Yes	1.6	1.2-2.0	<.001
Cath procedure time not recorded	0.9	0.62-1.2	.35
Contrast used >6 mL/kg (vs no)			
Yes	2.8	2.3-3.4	<.001
Contrast not recorded	0.5	0.36-0.8	<.001
Airway management (vs other)			
Intubated or tracheostomy	1.3	0.99-1.8	.061
Not recorded	3.3	1.6-6.7	<.001
Sedation (vs other)			
General anesthesia	1.5	1.1-2.1	.016
Not recorded	2.7	1.4-4.9	.002
Operator years since training (vs 5-24)			
<5 y	1.8	1.2-2.7	.005
≥25 y	1.7	1.0-2.8	.041
Operator cath volume (vs 100-200)			
<100 cases/y	1.2	0.85-1.8	.28
>200 cases/y	1.9	1.2-2.8	.003
Free-standing children’s hospital	1.4	0.97-2.0	.072
Hospital cath volume (vs 250-400)			
<250 cases/y	1.0	0.67-1.5	.96
>400 cases/y	1.4	0.88-2.1	.17

Odds ratios and 95% CIs are estimated from GEE models which cluster on the operator.

AE, adverse event; GEE, generalized estimating equation.

**Table 5:** Multivariate analysis for outcome days from procedure to discharge ≥2 days.

Variables	Odds ratio	95% CI	*P* value
Age at catheterization <1 y	2.29	1.9-2.8	<.001
Diagnosis single ventricle	1.48	1.2-1.8	<.001
Cardiac surgery within 90 d prior	1.31	0.99-1.7	.057
Hemodynamic vulnerability score (vs 0)			
1	1.19	0.97-1.5	.094
2	1.79	1.4-2.3	<.001
≥3	2.43	1.7-3.4	<.001
PREDIC^3^T risk category (vs 1)			
2	0.80	0.64-1.0	.051
3	1.30	1.0-1.6	.028
4	1.57	1.2-2.1	.002
5	1.48	1.1-2.1	.023
Any levels 3bc, 4, or 5 AE	5.93	4.6-7.6	<.001
Cath procedure time above threshold	1.53	1.2-1.9	<.001
Contrast used >6 mL/kg	1.28	1.0-1.6	.021
Operator years since training (vs 5-24)			
<5 y	1.51	1.2-2.00	.003
≥25 y	1.68	1.3-2.1	<.001
Operator cath volume (vs 100-200)			
<100 cases/y	1.18	0.83-1.7	.37
>200 cases/y	1.71	1.2-2.4	.003

Odds ratios and 95% CIs are estimated from GEE models which cluster on the operator.

AE, adverse event; GEE, generalized estimating equation.

### Discussion

This multicenter retrospective cohort study demonstrated that prolonged LOS occurs infrequently after outpatient PCCL procedures, with more than 90% of elective cases being discharged the same or the next day after the procedure. Using a prolongation in LOS as a marker of procedural harm cannot be interpreted in isolation without evaluating the predictors of a prolonged LOS. In this study, longer LOS after outpatient PCCL procedures was associated with a subset of factors that have previously been described to be independent predictors of high-severity AE (case type, infants or low weight, hemodynamic vulnerability, operator experience, and case time), and of all factors evaluated, a higher incidence of high-severity AE itself had the highest odds ratio for a prolonged LOS. A prolonged LOS was also associated with a higher volume of contrast, operator volume >200 cases/y, as well as single ventricle physiology, which have thus far not been described to be independent risk factors for AE.

Differentiating between AE and the potential for resultant harm is important because it highlights that not all AE have the same effect on patients. Moreover, it highlights a novel avenue to reduce harm. Previous efforts focused on reducing the incidence of AE (or specific subtypes).^13^^,^^14^ The progression from an AE to a catastrophic AE (or any adverse outcome that persists beyond the realm of the catheterization laboratory) on the other hand may be modified through QI efforts.^5^ Prolonged LOS after elective catheterization is a potential metric that reflects this. It is objective and can be measured from existing data sources. Importantly, it also has a higher incidence than catastrophic AE which is statistically expedient. Future research and QI efforts need to demonstrate that prolonged LOS is modifiable and ultimately leads to improvements in the outcome of patients. Regardless, the current work demonstrates the potential of a metric that quantifies durable harm beyond the PCCL procedures.

Interestingly, there was a notably higher proportion of cases that had a valvuloplasty related higher severity AE during the procedure in the patient population that stayed 2 days or longer. This could have been due to a variety of reasons, such as hemodynamic compromise following the procedure that requires stabilization prior to discharge or that obligates surgical repair during the same admission, (eg, severe aortic regurgitation following aortic valvuloplasty). Another example would be postprocedural arrhythmias that require initiation of pharmacotherapy. Although less common, it could also be related to elective surgical repair during the same admission for a procedure that has not resulted in an adequate physiologic result.

Occurrence of a high-severity (level 3bc,4 or 5) adverse event (HSAE) was associated with the largest increase in likelihood of prolonged LOS. It was not surprising, therefore, that almost all other independent predictors identified for an increased LOS mirrored those predictors that have previously been identified to be associated with an increased incidence of higher severity AE, such as age <1 year (or low weight),^2^^,^^9^^,^^15^ a higher hemodynamic vulnerability score,^2^^,^^9^ a higher PREDIC^3^T risk category,^3^^,^^7^ operator experience of either <5 or ≥25 years,^16^ as well as a prolonged procedure time.^8^ Although some patients with higher severity AE were discharged the same or the next day, there clearly is a big difference between cases without AE (93% discharged within 1 day), and those with any level 3bc/4/5 AE (only 60% discharged within 1 day). This underscores the importance of these AE and their potential significance to the patients. An important question is how prolonged LOS complements this. The added value of increased LOS is that it is more specifically related to harm extending beyond the catheterization laboratory than the recorded AE, highlighting both situations where the high-severity AE does and does not lead to harm, situations in which harm occurred even without an objective AE recorded during the procedure, and situation in which a lower severity AE does lead to harm.

As such, the same considerations apply to LOS, that were previously discussed when analyzing the impact of those factors on the incidence of AE. For example, it has previously been reported (and discussed) that the fact that very senior operators have a higher incidence of AE is likely related to the fact that those operators get referred complex cases, where the risk-adjustment methodology is not granular enough to account for subtle nuances that can characterize an increased procedural complexity and risk within the same procedure type.^16^ Similar considerations apply when discussing resultant prolongation of hospital stay.

The only predictors for an increased LOS that have not previously been described as predictors for HSAE, included use of contrast >6 mL/kg, operator volume >200 cases/y, and the presence of single ventricle physiology. Operator volume was evaluated previously though, with a trend of low volume operators (<150 cases a year) having a lower incidence of HSAE.^16^ This may be related to the fact that a volume of at least 200 cases a year reflects a conservative floor for “busy interventionalists” who may perform a higher percentage of complex procedures (not necessarily captured within the procedure type risk group) when compared to interventionalists who perform only a smaller number of cases each year. The increased incidence of a longer LOS with higher use of contrast, may reflect to some extent the increased procedure length, and possible secondary effects on the incidence of HSAE. Single ventricle patients though, fall into a different category and the most likely explanation for a higher incidence of a prolonged LOS is the fact that these patients often have significant other associated medical problems (such as the need for nutritional support), and frequently inpatient admissions facilitate addressing these issues at the same time as the cardiac catheterization procedure, which as a result can prolong the hospital stay, even in the absence of an AE. Furthermore, unexpected cardiac catheterization findings in single ventricle patients who have not yet undergone a Glenn procedure may be more likely to lead to surgical intervention during the same admission. In addition, some single ventricle patients may be electively admitted with the specific plan to perform cardiac catheterization and then directly proceed to the next stage of surgery within a couple of days. These data though are important to incorporate into procedural planning when considering elective cardiac catheterization for patients with single ventricle physiology.

Interestingly, 7% of patients without any AE in the cardiac catheterization laboratory, had a prolonged LOS of 2 days or more. This may of course reflect similar issues as described for single ventricle patients (additional medical needs, planned surgery post catheterization), but may also signify that some patients may experience adverse outcomes after the procedure that lead to a prolonged LOS, even without any recorded AE in the cardiac catheterization laboratory. Examples include patients after balloon aortic valvuloplasty who show ventricular arrhythmias during postprocedural recovery and subsequently require treatment with beta blockers, or patients with single ventricle or complex 2-ventricle physiology, who demonstrate lower-than-expected oxygen saturation during recovery. Furthermore, some patients may have minimal symptoms of a viral illness preprocedure that impacts recovery from anesthesia and requires an additional in-hospital stay.

Conversely, patients with even higher severity AE may sustain no harm beyond the catheterization laboratory and are discharged within 24 hours. An example would be a patient who requires defibrillation due to a wire induced ventricular arrhythmia but recovers immediately and is discharged the next day. This is where it becomes important to distinguish between AE in the catheterization laboratory (which may not necessarily result in any harm to the patient), and actual harm or adverse outcome to a patient, sometimes even after an otherwise uneventful procedure without any documented AE, which is where the metric of increased LOS provided added value beyond AE recorded in the catheterization laboratory.

Of note, there was a wide variation between centers in the percentage of patients who stayed 2 days or more, ranging between 1.9% and 12.6%. Possible explanations include differences in the threshold to perform surgery during the same admission as the elective cardiac catheterization, which is unlikely though to account for those differences. Although specific policies requiring a certain duration of an admission beyond day 1 postprocedure usually do not exist, it is likely that centers and operators may have different thresholds of observing patients postprocedure who have either sustained an AE or have shown postprocedural changes (such as lower saturations) that were not expected.

### Limitations

This study has several limitations. As is the case for most registry-based studies, there remains a risk of potential manual data entry errors, which cannot be eliminated completely with random data entry validation and data audit. However, for the most part, these errors are unlikely to be of a systematic nature that would skew results in one direction or another.

Most importantly, as discussed above, prolonged LOS is not always the result of an AE and may not always be related to the cardiac catheterization procedure itself. For instance, some patients undergo cardiac catheterization with a specific plan to pursue surgery or other medical care during the same admission, or catheterization reveals an issue unrelated to the procedure that requires hospitalization. Expanding data capture to identify patients who underwent elective cardiac surgical procedures or other procedures following the same admission or those who required planned noncardiac medical care, would solve this problem and should be a change that could easily be implemented in registry data capture. Equally, metrics that had better attributability would be superior, but as described in previous sections, they are not ascertainable using existing data sources.

There is also the potential that prolonged LOS is a low-bound estimate of harm. Some patients discharged the same or next day, may experience harm from events that were missed prior to discharge (such as vascular occlusion). Additionally, this metric necessarily is limited to outpatient catheterizations and does not address harm that occurs in inpatients, where prolongation of hospital stay will be all but impossible to ascertain. Finally, there is an inevitable risk of unmeasured confounding and type II error, even in a large multicenter registry that has worked to capture factors that influence the risk of AE.

## Conclusion

We conclude that prolonged LOS is a potentially useful metric following outpatient PCCL procedures, which may be a complement to existing safety measures. Clearly this is only the first step of many to fully evaluate the utility of LOS as a potential measure of procedural harm and account for a variety of potential cofounding variables that registry data were unable to assess. Additional research is necessary to demonstrate that prolonged LOS is modifiable and ultimately improves overall outcome, but the current research demonstrates the potential of studying the process(es) that lead from an AE in the PCCL to durable harm.

Beyond providing an additional metric for procedural harm, this study has also identified the presence of single ventricle physiology as an additional independent factor associated with a prolonged LOS, which needs to be considered in procedural planning and (bed) resource allocation.
